# Spiritual Care During COVID-19: An Exploratory Study of the Experience of Hospital Chaplains in the Amazon Region

**DOI:** 10.1007/s10943-026-02652-z

**Published:** 2026-04-28

**Authors:** Luiz Francisco Rocha e Silva, John Lennon Moura Lima, Ianna Laisa Souza Sirqueira, Fábio Marcon Alfieri

**Affiliations:** 1 Hospital Adventista Manaus, Manaus, Amazonas Brazil; 2 Mestrado em Promoção da Saúde, UNASP, São Paulo, Brazil

**Keywords:** COVID-19 pandemic, Hospital chaplains, Spiritual care, Multidisciplinary team, Hospitalized patients

## Abstract

The COVID-19 pandemic brought about a scenario of physical, social, and emotional illness that significantly increased the need for spiritual care, particularly in hospital environments, where individuals were more vulnerable, fragile, and anxious. In this context, the hospital chaplain played a strategic role within the multidisciplinary care team for hospitalized patients, serving as the primary provider of spiritual care. This article presents an experiential report of the role of hospital chaplains in providing spiritual support to the multidisciplinary team, patients, and their families in a nonprofit, religiously affiliated hospital in the Brazilian Amazon during the COVID-19 pandemic. The emergence of the coronavirus in the hospital led to decisions that reoriented care routines to ensure quality services and the safety of both patients and staff, significantly impacting the chaplaincy service’s provision of spiritual support. Like other healthcare professionals, chaplains adapted their practices. Notable changes included restricted access to patients and family members, the elimination of physical contact, continuous use of personal protective equipment (PPE), and strict adherence to hygiene protocols. Visits became shorter and required greater preparation, aiming to ensure safety for all. The chaplaincy’s collaboration with the multidisciplinary team was essential in providing emotional and spiritual assistance. Through worship services, prayers, active listening, and a welcoming presence, chaplains supported professionals in facing fear, exhaustion, and grief, thereby strengthening resilience. Ultimately, spiritual care became a key pillar for collective recovery, fostering hope, meaning, and daily renewal amid the crisis.

## Introduction

The COVID-19 pandemic, caused by the SARS-CoV-2 virus, was first identified in Wuhan, China, in December 2019. It became a significant global public health crisis in February 2020, resulting in 775 million confirmed cases and over 7 million deaths worldwide (WHO, 2024).

At the beginning of the COVID-19 pandemic in Brazil, Northern Brazil, particularly the state of Amazonas, received widespread media attention due to low adherence to government social distancing measures. These behaviors aggravated social disparities, contributing to high infection rates in the region. Furthermore, limited hospital capacity, characterized by fewer intensive care units (ICUs), fewer beds, and fewer ventilators than in other areas of the country, exacerbated the situation in both the public and private sectors (Lopes et al., 2024). As a result, Amazonas recorded the highest incidence of positive cases in Brazil (4,474.6 per 1,000,000), overwhelming the local healthcare system (de Souza Vieira et al. 2023).

Hospitalization due to SARS-CoV-2 infection resulted in considerable emotional strain for patients and their families. Strict isolation protocols, uncertainty about prognosis, and the severity of the illness intensified mental distress, directly affecting the emotional well-being of hospitalized individuals (Byrne & Nuzum, 2020; Vahabi et al., 2025). Studies have shown that COVID-19 inpatients reported high rates of insomnia, somatization, and fear, as well as correlations between emotional disorders—such as depression and anxiety symptoms—and prolonged hospital stays (Lee et al., 2023).

In this context of increased physical, social, and emotional suffering, spirituality and religious practices emerged as protective factors not only for mental health but also for physical well-being (Coppola et al., 2021; Zhu et al., 2023). In disaster and pandemic contexts, spiritual care involves a holistic assessment of the patient, acknowledging personal values and beliefs, identifying spiritual needs, and providing appropriate support. Such practices foster effective coping and promote comprehensive well-being (Byrne & Nuzum, 2020; de Diego-Cordero et al., 2022).

## Literature Review

Research has shown that many patients express a desire for spiritual care, even when it is not directly tied to traditional religious practices. Under these circumstances, hospital chaplains play a central role as trained professionals, providing guidance and comfort in high-risk situations, end-of-life care, and ethical dilemmas related to faith and medical decision-making. (Connolly et al., 2022; Kirchoff et al., 2021; Kuepfer, 2022).

According to Koenig (2020), chaplaincy services are regarded as essential in hospital settings, particularly for healthcare providers facing religious or existential challenges. Chaplains fulfill an essential role in helping patients address conflicts and concerns that, if unresolved, may negatively impact their physical health, emotional state, and overall well-being.

Fear of death, isolation, insecurity, and moral distress intensified the need for emotional and spiritual support among patients, families, and healthcare professionals (Colorafi et al., 2025; Esperandio, 2020; Guimarães-Teixeira et al., 2023). Despite access restrictions and reduced in-person visits, chaplains became increasingly sought after, providing comfort, hope, and meaning amid loss and uncertainty. This context enhanced the visibility of spiritual care as an essential component of holistic treatment, underscoring its importance for psychological well-being and resilience during times of crisis (Lee et al., 2023; Snowden, 2021; Wierstra et al., 2020).

Restrictions on patient access, social isolation, fear of the unseen pathogen, and strict infection control protocols greatly limited close interpersonal interactions for both chaplains and healthcare professionals, transforming the traditional dynamics of spiritual care in hospital settings (Desjardins et al., 2021; Domaradzki, 2023; Kwak et al., 2022; Tracey et al., 2023). Nevertheless, while the public health crisis constrained chaplains’ physical presence and routine practices, it simultaneously underscored the unique value of hospital chaplaincy in promoting holistic care. Recent studies highlight that, despite such limitations, chaplains remained essential figures, providing spiritual and emotional comfort during one of the most critical periods in modern healthcare history (Domaradzki, 2022; Kirchoff et al., 2021; Lazzarino & Papadopoulos, 2023).

Although there is extensive international literature on spirituality and religiosity during the COVID-19 pandemic, few studies have specifically examined the challenges faced by hospital chaplains in the South American context, particularly in Northen Brazil. Little is known about the adaptations these professionals made or how the health crisis affected the delivery of spiritual care in faith-based hospitals. Understanding these experiences is essential for enhancing institutional resilience and improving chaplaincy preparedness for future public health emergencies.

Given the context, this study aims to present an experiential report on the role of hospital chaplains during the COVID-19 pandemic, focusing on the delivery of spiritual care to patients, their families, and multidisciplinary teams at a nonprofit, religiously affiliated hospital in the Brazilian Amazon.

## Methodology

This study is an experiential report based on the actions of the Chaplaincy Service of a nonprofit, faith-based hospital which is part of the Adventist Health Network, located in Manaus, Amazonas, Brazil. The hospital has approximately 140 beds and offers comprehensive medical services, including intensive care units (ICUs), emergency care, outpatient clinics, and diagnostic services.

The reflections presented here are drawn from the experiences of four hospital chaplains who were directly involved in providing spiritual care to the multidisciplinary team, hospitalized patients, and their families during the COVID-19 pandemic, from March 2020 to March 2022. During the period in which the events described occurred, four chaplains were employed in the hospital chaplaincy service, and all of them were included in the study. Therefore, the response rate was 100% (n = 4/4), as no additional chaplains were working in the institution at that time. Table 1 shows the demographic profiles of the participants.

**Table 1:** Participant demographic characteristics (N = 4)

Participant	Age	Gender	Employment status	Years of chaplaincy experience	Education	Religious affiliation
C1	27	Male	Full time	2 year	Bachelor in Theology	Seventh-day Adventist
C2	31	Male	Full time	2 years	Bachelor in Theology	Seventh-day Adventist
C3	35	Male	Full time	1 years	Bachelor in Theology	Seventh-day Adventist
C4	39	Male	Full time	1 year	Bachelor in Theology	Seventh-day Adventist

The chaplains’ experiences during the pandemic were gathered through group meetings, where they shared and reflected on their activities. The authors subsequently synthesized these reflections into consensual themes. The selected topics represent the aspects that most significantly impacted chaplaincy work during the pandemic and align with themes found in the international literature on spiritual care in times of health crisis. Representative quotes from the chaplains have been incorporated into the narrative of each thematic section to support the experiential account.

The analytical approach employed in this experiential report involved a descriptive synthesis of the actions carried out by the hospital Chaplaincy Service. This was complemented by a thematic analysis of the chaplains’ reflections (Braun & Clarke, 2006). The aim was to identify the main challenges, adaptive strategies, and the impact of spiritual care throughout the pandemic.

According to Minayo (2010), experience reports allow for the systematization of practical knowledge, enabling critical reflection on actions taken in real-world contexts and contributing to the development of new knowledge in the field of health.

The present study is part of the project “Hospital Chaplaincy in Comprehensive Patient Care in a Private Health Network,” which was reviewed and approved by the Research Ethics Committee of the Adventist University Center of São Paulo (UNASP), under protocol CAAE: 67,917,823.4.0000.5377, with approval number 6.013.641. This article adheres to all international ethical guidelines outlined in the Declaration of Helsinki (World Medical Association, 2013) and Resolution No. 510/2016 (Brazil, 2016) of the National Health Council.

## Experiential Report and Discussion

This experiential report is organized into thematic sections, each beginning with a narrative account of events as experienced by the chaplains who authored the report, followed by a literature-based discussion of each topic, *namely: (1) Spiritual Care Before the Pandemic, (2) The Onset of the Pandemic and Hospital Adaptations, (3) Changes in the Provision of Spiritual Care by the Chaplaincy, (4) The Brave New World, (5) Spiritual Care Provided to the Multidisciplinary Team, (6) Death and Spiritual Care, and (7) Self-Care Among Hospital Chaplains.*

1. Spiritual Care Before the Pandemic

The hospital chaplaincy team has long conducted bedside visits with patients of varying clinical complexity. These visits fostered close relationships with patients, often extending to family members and building trust—an essential component of meaningful spiritual care. Prayers, hymns, and physical touch, such as handholding, were common elements of these interactions. Pastoral staff members provided support in individual patient rooms, shared wards, intensive care units, surgical suites, and even outpatient reception areas, always adhering to the patient safety protocols in place at the time. It is possible to observe this dynamic in the following chaplains’ statements:

Before the pandemic, spiritual care was mainly carried out through more direct contact with patients and their families, especially at the bedside. This face-to-face encounter allowed for a much more sensitive connection… (C2, 2022).

Our main work was visiting patients. We spent a lot of time talking with them, building relationships and having very close contact. (C3, 2022).

Support for the spiritual needs of the hospital extended beyond patients to include the multidisciplinary staff. Chaplains regularly visited nursing stations to lead prayers, share biblical messages, and sing hymns with the healthcare teams.

Spiritual care providers serve as strategic professionals in comprehensive healthcare, offering pastoral assessments, emotional support, counseling, and guidance in ethical matters. Their presence helps alleviate stress among patients, families, and healthcare teams, promoting more humane and holistic decision-making that considers not only physical health but also emotional, cultural, and spiritual dimensions (Desjardins et al., 2023; Earl et al., 2022; Tracey et al., 2023).

Timmins et al. (2018), emphasize that the core mission of any hospital—and of its chaplaincy team—must be to provide spiritual and religious care to patients and, when appropriate, to their families. In Brazil, this right is protected by federal legislation. Law No. 9.982 of 2000 that regulates the provision of religious assistance and states:

["Art. 1. Religious ministers of all faiths are guaranteed access to public or private hospitals, as well as to civil or military correctional institutions, to provide religious assistance to inpatients, provided this is in agreement with the patient or, in the case of patients no longer in full possession of their mental faculties, with their family members.**"**] (Brazil, 2000).

Koenig (2020), emphasizes that the chaplain’s support is crucial when religious or spiritual questions arise, enabling patients to confront spiritual dilemmas that, if left unaddressed, may negatively impact their health and well-being. In addition to supporting patients and their families during critical moments, hospital chaplains are also tasked with caring for their colleagues on the multidisciplinary healthcare team—an essential component of their professional practice (Culmann et al., 2024; Knapp et al., 2025).

(2) The Onset of the Pandemic and Hospital Adaptations

The COVID-19 pandemic reached the city of Manaus in March 2020, bringing with it uncertainty within the scientific community regarding how to address a novel and highly impactful disease. The Manaus Adventist Hospital, one of the largest private hospitals in the region and certified to international quality standards, became a key referral center for patient care during the pandemic. The hospital’s multidisciplinary care team includes physicians, nurses, physical therapists, pharmacists, psychologists, dentists, nutritionists, speech therapists, nursing technicians, and hospital chaplains.

Hospital operations were immediately impacted by the pandemic, prompting decisions to restructure daily routines and ensure the maintenance of quality care and the safety of both patients and staff. Initially, several staff members, including chaplains, were temporarily removed from their duties for safety reasons. As a result, in-person spiritual care services were suspended. Two chaplains reported as follows:

It was a time when people really needed attention, because it was an unknown disease. People needed that spiritual care, that support, and we were unable to provide it. (C1, 2022).

During the pandemic, the chaplaincy team was temporarily removed from in-person activities in accordance with institutional guidelines…Over time, the multidisciplinary team itself began to recognize the importance of chaplaincy on the front line and asked for us to return. (C2, 2022).

The chaplains reported that the multidisciplinary team felt the absence of spiritual support, both for themselves and for patients and their families. Consequently, a formal request was made for the chaplains to return to their duties, and they were reinstated less than two weeks after the initial suspension.

The hospital experienced a surge in patient volume and a high rate of viral transmission. Many staff members became infected and were required to be isolated for 15 days or more. Various adjustments were implemented in patient flow and the hospital’s physical infrastructure, including the construction of physical barriers between units and the reorganization of services. Notable changes included converting a surgical ward to a COVID-19 clinical unit, admitting patients directly into the emergency department, transforming a clinical unit into a step-down care unit, and suspending elective surgeries.

These adaptations were also observed in other Brazilian hospitals. Tavares et al. (2024), describe how a hospital in the state of Rio de Janeiro restructured its standard operations to meet the conditions imposed by the pandemic. The interventions implemented encompassed bed management, human resources (including reorganization, safety, and mental health support), supply chain logistics, and infrastructure. In another institution, Rodrigues and Silva (2020), reported that workers considered at risk, such as the elderly and those with chronic illnesses, were reassigned to other departments or placed on leave.

Throughout Brazil, healthcare professionals working in hospital, had to adapt quickly in order to maintain the quality of care. For instance, nurses adopted more stringent infection prevention and control protocols and were required to wear personal protective equipment (PPE) continuously (De Paula et al., 2023). Nutrition teams were also affected, primarily due to staff shortages, which led to adjustments in meal planning and variations in the number of meals prepared, depending on the specific needs of each facility (de Oliveira Sampaio, 2023).

(3) Changes in the Provision of Spiritual Care by the Chaplaincy

The most significant changes experienced by the chaplaincy team included restrictions on chaplains’ visits to patients, limitations to family access, a prohibition on physical contact, changes to isolation protocols, and a sharp increase in the number of patients in isolation.

During the pandemic, chaplains contracted COVID-19 at different times. When one chaplain was placed on leave due to infection, the increased workload was absorbed by those who remained on duty. However, upon returning from illness, they reported feeling more confident and immunologically better prepared to care for patients and families. Additional attention was given to hand hygiene and to the proper procedures for donning and doffing PPE, including caps, masks, and gowns.

Chaplaincy interventions were carried out through bedside visits. Although patients were free to decline spiritual care, the approach was well received by individuals from a variety of religious backgrounds, including those with no religious affiliation.

During visits, the physical contact with patients and their families was completely suspended. Preparing for each visit required more time, while the duration of the visit itself was shortened to reduce the risk of infection. Nevertheless, the quality of care was maintained. Chaplains noted how these spiritual interventions helped to reduce patient stress and anxiety. Expressions of faith and hope were experienced as a kind of “medicine,” as confirmed by feedback from the multidisciplinary healthcare team.

At times, chaplaincy services were delivered remotely to patients and their families. Spiritual care was then offered via video calls and phone conversations. All protocols established by the government’s Department of Health were strictly followed, as affirmed by chaplains C4 and C2:

We spent a lot of time putting on protective equipment and taking all the precautions, for our safety and also for the patients. I spent more time getting ready than actually visiting. But I followed all the rules. (C4, 2022).

In this situation, we had to reinvent ourselves, looking for alternatives to keep providing spiritual care, like using digital devices for remote support. (C2, 2022).

In Table 2, the adaptations in spiritual care practices by hospital chaplains before and during the COVID-19 pandemic are summarized.

(4) The Brave New World

**Table 2:** Key adaptations in spiritual care practices by hospital chaplains before and during the COVID-19 pandemic

Aspect	Before the pandemic	During the pandemic
Care environment	All hospital settings, including ICU, operating room, and outpatient clinics	Primarily emergency room, inpatient units, and ICU; some restricted areas
Patient interaction	Physical and emotional proximity to patients and families	Mandatory physical distancing
Physical contact	Common physical contact (hugs, handshakes, holding hands during prayers)	Physical contact completely suspended
Spiritual interventions	Prayers, hymns, Bible readings, emotional support	Prayers, biblical promises, instrumental music, active listening
Family access	Regular family access to patients	Severely restricted or prohibited
Protective equipment use	No continuous use of PPE	Continuous use of PPE (mask, cap, gown)
Preparation and duration of visits	Rapid preparation; longer visits with extended presence	Lengthy preparation; shorter visits to reduce exposure risk
Support for healthcare team	Devotional services and prayers with the staff at nursing stations	Spiritual and emotional support for the team under pressure
Use of technology	Not required	Video calls, phone calls, and recorded messages
Collaboration with care team	Regular and integrated presence	Spiritual care frequently requested by other healthcare professionals
Chaplain’s emotional and spiritual resilience	Supported by routine, regular spiritual practices, and peer interaction	Increased emotional burden; greater need for self-care, prayer, psychological support

The “new world” created by the COVID-19 pandemic imposed new rules and redefined patterns of behavior and interpersonal interaction. In a very short time, the demands for adaptation became extreme. Spirituality contributed significantly to how individuals make sense of this experience, as it addressed the human dimension of the “meaning of life.” Humans have a deep need to find meaning in their experiences—especially in those marked by suffering, such as the events brought on by COVID-19 (Byrne & Nuzum, 2020; Esperandio, 2022). This perception also changed the chaplains’ approach, as reflected in the statement of chaplain C2:

From that moment on, we noticed a significant change in how we approached spiritual care. We still talked about things like forgiveness, love, and hope, but there was a much deeper focus on helping people find meaning, deal with suffering, and face the reality of death. (C2, 2022).

Like the present report, chaplaincy services around the world adapted to diverse contexts and communities. These services were able to provide emotional, spiritual, and religious support during a period marked by strict limitations on patient access and the widespread suspension of religious gatherings (de Diego-Cordero et al., 2022; Drummond & Carey, 2020; Siesage et al., 2023).

An international survey of chaplains from 36 countries, including South American countries such as Chile and Colombia, revealed a substantial disruption in their usual practices during the pandemic, primarily due to the enforcement of social distancing. As a result, chaplains increasingly relied on technology to stay in contact with patients and families, and many redirected their efforts toward supporting healthcare staff (Snowden, 2021). Byrne and Nuzum (2020) also reported the use of virtual video-call technology in a hospital in Ireland to maintain connections between patients and their loved ones. This intervention had a significant positive impact on reducing anxiety, fear, and uncertainty among both patients and their families.

In Chile, chaplains provided spiritual care to hospitalized patients with severe COVID-19 and their families during pandemic restrictions through the delivery of interdisciplinary telehealth services (Palma et al., 2021). These aspects were also observed in the study by Tata et al. (2021), which included 1,657 chaplains from various continents, including South America.

In the United States, studies by Kwak et al. (2022) and Desjardins et al. (2023), have shown that the challenges posed by COVID-19 have intensified social isolation and increased concerns about the mental health of patients, families, and healthcare providers, profoundly impacting chaplains’ roles. These professionals were compelled to develop safer and more creative ways to provide spiritual care, even amid severe restrictions, such as limited access to hospital units and the inability to rely on non-verbal communication.

Consistent with the experiences reported by the chaplains in this report, previous research has highlighted that the most deeply felt loss was the lack of face-to-face interaction, physical contact, and social connection. The inability to perceive facial expressions—such as smiles and other emotional cues—created a profound sense of disconnection. The lack of genuine, warm human encounters made it harder for chaplains to emotionally engage, limiting their ability to express the spontaneity and depth of presence that are central to effective spiritual support. Simple gestures like handshakes, hugs, or holding a patient’s hand during prayer are powerful forms of communication, especially for those with verbal communication challenges (Byrne & Nuzum, 2020; Desjardins et al., 2021, 2023; Kuepfer, 2022).

Other studies in Europe (Coppola et al., 2021; Papadopoulos et al., 2021; Siesage et al., 2023; Stirling, 2021; Wierstra et al., 2020), Australia (Drummond & Carey, 2020; Jones et al., 2022) and Canada (Kuepfer, 2022) have a similar experience as it was reported here; showed that spiritual support for patients, families, healthcare teams, religious leaders, and chaplains themselves had to be significantly scaled back, both in terms of quality and scope. Much of this care was delivered through technological means or symbolic gestures in domestic settings.

(5) Spiritual Care Provided to the Multidisciplinary Team

Hospital chaplaincy service also provided spiritual care to hospital staff. Multidisciplinary providers also experienced emotional fragility in the face of numerous patient deaths, overwhelming numbers of critically ill individuals, and constant exposure to infection risks—leading to fear not only for their own lives but also for the safety of their families. The chaplains reported this interaction with hospital staff as follows:

We also noticed this tension about what to expect from the disease, even among healthcare professionals. Even though they were used to dealing with many different situations in healthcare, they were worried about their own families who were at risk. They often talked about their elderly parents. (C1, 2022).

I supported doctors, nurses, and other professionals who were emotionally shaken from dealing with so many critically ill patients and so many deaths like they had never seen before. (C3, 2022).

The spiritual care offered by chaplains helped the multidisciplinary team reframe their experiences with each patient. Beyond clinical staff, chaplains also provided care to administrative personnel, who likewise felt physically, emotionally, and spiritually vulnerable. Even if indirectly, all hospital employees were part of the broader network of care supporting patients and their families.

Recent studies have shown that healthcare professionals—especially those on the front lines during the COVID-19 pandemic—experienced high levels of vulnerability and psychological disorders, including depression and anxiety (Colorafi et al., 2025; Guimarães-Teixeira et al., 2023; Stirling, 2021; Zhu et al., 2023). Post-traumatic stress symptoms were associated with anxiety about the death of older adults, insufficient personal protective equipment, and repeated exposure to death, according to a study involving healthcare professionals from four Latin American countries (Abeldaño Zuñiga et al., 2021). Corresponding emotional difficulties were reported by hospital staff in this study.

The literature suggests that, in the face of the numerous challenges brought on by the pandemic, spirituality emerged as a vital ally for healthcare professionals in managing the human dimensions of care, which include fear, anguish, and frustration (dos Santos Teixeira et al., 2021; Knapp et al., 2025). Spirituality was considered an effective coping strategy, fostering inner strength, resilience, and well-being, while also contributing to greater patient satisfaction with the care received (Byrne & Nuzum, 2020; de Diego-Cordero et al., 2022).

Many physicians and nurses turned to chaplains for emotional support, highlighting the value of this resource in helping them manage their own emotions. Healthcare providers recognized the essential role of them in hospital wards, especially during the crisis, and often requested their presence when spiritual needs were identified in patients (Domaradzki, 2022, 2023; Tata et al., 2021; Wierstra et al., 2020). Supporting the healthcare team became a central aspect of the chaplaincy’s work during the pandemic at the Manaus Adventist Hospital.

The spiritual care provided by chaplains to patients and families also helped reduce stress among the multidisciplinary team. This positive impact—although indirect—was frequently acknowledged by healthcare professionals, who reported feeling more supported in difficult situations.

When chaplains were present to accompany families during crises—in the Emergency Room, ICU, or during complex medical decisions or end-of-life care—their emotional support and compassionate presence helped foster a more balanced and humane care environment (Knapp et al., 2025).

(6) Death and Spiritual Care

The COVID-19 pandemic introduced a reality marked by an unprecedented scale of death within the hospital setting and in the experience of the chaplains involved in this report. The continuous flow of deaths deeply affected patients, families, healthcare professionals, and chaplains alike. In many cases, chaplains were present during the final hours of patients’ lives—often being the only human presence at the bedside when death occurred. They became silent witnesses to farewells without embraces and to tears that could not be wiped away. Frequently, they participated in restricted and silent funerals where families were absent due to strict health protocols. The statements of chaplains C2 and C4 summarize these experiences:

One of the biggest challenges of the pandemic was dealing with so many deaths. It’s hard to even describe the scale and the impact… More than the virus itself, what we saw most was the anguish related to facing death. (C2, 2022).

We were used to caring for patients at the end of life and their families, but not on the scale we experienced during the pandemic… The hardest part was seeing that patients and families couldn’t have their moments of goodbye because of the isolation. (C4, 2022).

The healthcare professionals were also deeply affected by the sheer volume of loss and sought the chaplain as a safe space to express their pain, fatigue, and need for meaning. The chaplain’s presence in the hospital became as anticipated as medication itself, often regarded as a form of soul therapy.

From the perspective of Polish hospital chaplains, Domaradzki (2023), emphasized that, while these professionals were accustomed to issues of death and dying, the COVID-19 pandemic profoundly altered their perception of mortality. Death became a routine part of hospital life on an unprecedented scale, with a marked increase in the number of critically ill patients. In the United Kingdom, Siesage et al., (2023), reported the emotional burden experienced by chaplains, including the challenge of managing multiple simultaneous deaths, moral distress, and feelings of helplessness in the face of overwhelming losses.

Desjardins et al., (2023), noted that, among American chaplains, it was common to encounter cases where patients died without family present or were visited only moments before death. Early in the pandemic, when knowledge about the virus was limited and PPE was scarce, many chaplains refrained from entering isolation rooms unless death was imminent.

Similarly, Murphy (2020), highlighted that, although COVID-19 caused many premature and unexpected deaths, it was the isolation and social distancing measures that had the most profound impact on funeral rituals and the mourning process. These restrictions significantly intensified the pain and suffering of grieving families. In the face of visible anguish on both sides, these professionals served not only as emotional support but also as mediators of compassionate care, facilitating moments of love, reflection, and spiritual connection (dos Santos, 2020).

(7) Self-Care Among Hospital Chaplains.

Although chaplains have always exercised caution to protect their own heath safety and that of their families, nothing compared to what they encountered during the COVID-19 crisis concerning infections. The emotional burden during the pandemic was also overwhelming. While chaplains were accustomed to dealing with difficult hospital situations—such as patient deaths, cancer diagnoses, and the suffering of parents facing their children’s illnesses—the emotional demands brought on by the pandemic were far more intense. The personal impact of these experiences is evident in the following statements:

I saw patients who were the same age as me in critical condition. And you start to put yourself in that situation, realizing that you are also vulnerable… I kept thinking about the elderly in my family, my parents, my relatives. It really affects you emotionally. (C1, 2022).

Sometimes we had to seek support from the hospital’s psychology service for ourselves. It was a way to relieve the tension and keep going. It was an emotional roller coaster. Taking care of our mental health was essential. Our connection with God, prayer, and reading the Bible were our main sources of strength. (C4, 2022).

Additionally, social isolation, fear of infection, and prolonged exhaustion demanded not only resilience but also new forms of self-care and emotional support from chaplains.

Their work transcended the professional sphere, encompassing their personal and spiritual lives. To remain grounded, many increased their daily spiritual practices, dedicating more time to prayer, meditation, and Bible study. They also turned to books on spirituality, theology, and personal development for inspiration and renewal.

When needed, they sought professional psychological support—an essential measure that helped reinforce their resilience and enabled them to navigate the emotional crises faced during the pandemic. Mutual support among chaplaincy team members, including open conversations and shared reflections, was also a crucial aspect of self-care.

According to Byrne and Nuzum (2020) and Desjardins et al. (2021), strict adherence to safety protocols—including proper use of PPE—created safer conditions for chaplains to maintain physical presence at the bedside. Complying with international guidelines not only protected their health but also ensured the continuation of effective spiritual care through listening, emotional support, and religious assistance, even in a context of extreme social restriction. Even when distanced from traditional pastoral practices, chaplains sought to maintain the authenticity of their support, showing creativity in how they offered spiritual presence and comfort, transforming moments of vulnerability into opportunities for resilience and hope.

Despite the challenges, many chaplains reported significant positive outcomes during the pandemic, such as increased institutional recognition of the value of spiritual care and of their own role within the hospital setting (Desjardins et al., 2021; Domaradzki, 2022; Wierstra et al., 2020). Their commitment to their pastoral duties remained strong, even when some activities were restricted, reinforcing their sense of calling and personal mission. By tending to their own physical, mental, and spiritual health, chaplains preserved their capacity for service and made a decisive contribution to a comprehensive model of care for hospitalized patients during the public health crisis.

## Limitations

This study presents limitations inherent to the experiential report methodology, as it is based on the specific experiences of hospital chaplains in a single institution, which may restrict the generalizability of the findings to other healthcare settings. Moreover, the observations and reflections are influenced by the authors’ subjectivity. The study also relied on self-reported, retrospective data, which may be affected by recall bias or confirmation bias, as participants reflected on emotionally charged experiences during the COVID-19 pandemic. Additionally, the small and context-specific sample—comprising four chaplains from a single faith-based hospital—limits the scope of applicability to broader and more diverse institutional contexts. Despite these limitations, the report offers a valuable contribution to understanding the role of spiritual care during public health crises. It may inform future research using more rigorous and complementary methodological designs.

## Conclusion

The present experiential report from hospital chaplains during the coronavirus pandemic highlights the essential role of spiritual care as part of integrated health support for hospitalized patients, their families, and the multidisciplinary team. Although the COVID-19 crisis limited the scope and nature of spiritual care in hospitals, the chaplains featured in this report remained steadfast in fulfilling their responsibilities as providers of spiritual support within the clinical setting.

Like other healthcare professionals, chaplains had to adapt their practices. Key adjustments included restricted access to patients and their families, elimination of physical contact, continuous use of PPE, and stricter hygiene protocols. Visits became shorter and required additional preparation, all aimed at ensuring safety for both staff and patients. Thus, hospital chaplains need to be prepared and open to developing strategies for delivering spiritual care in challenging situations, such as those encountered during the COVID-19 health crisis.

The chaplaincy’s involvement with the multidisciplinary team during the pandemic was essential in providing both emotional and spiritual support. Through worship, prayer, active listening, and a comforting presence, chaplains helped healthcare workers face fear, exhaustion, and grief—ultimately strengthening their resilience. Spiritual care became a foundational element of collective recovery, fostering hope, meaning, and daily renewal amid the crisis.

This experiential report, along with other studies in the literature, provides a foundation for strengthening hospital chaplain training programs and enhancing the capacity of healthcare professionals to deliver spiritual care. Furthermore, it may inform the development of institutional and governmental policies that promote the ethical and structured integration of spiritual care into clinical practice.
